# Vitamin C and Vitamin E Protected B_95-8_ and Balb/c-3T3 Cells from Apoptosis Induced by Intermittent 50Hz ELF-EMF Radiation

**Published:** 2017-01

**Authors:** Zhen DING, Jintao LI, Fan LI, Mohammadreza Mohammadzad MEPHRYAR, Shuicai WU, Chen ZHANG, Yi ZENG

**Affiliations:** 1. Beijing Key Laboratory of Environmental and Viral Oncology, School of Life Science and Bio-Engineering, Beijing University of Technology, Beijing, 100124, China; 2. State Key Laboratory for Infectious Disease Prevention and Control, National Institute for Viral Disease Control and Prevention, Chinese Center for Disease Control and Prevention, Beijing, 100052, China; 3. China Academy of Telecommunication Research of Ministry of Industry and Information Technology, Beijing, 100191, China

**Keywords:** Extremely low frequency, Apoptosis, Vitamin C, Vitamin E

## Abstract

**Background:**

The extremely low-frequency electromagnetic field (ELF-EMF), mainly emitted by electric transmission lines and household electronic appliances, is becoming a worldwide health risk. It is imperative to investigate the biological impacts of ELF-EMF and to identify products that are resistant to the radiation from 50 Hz ELF-EMF. In this study, we investigated the biological impacts of apoptosis caused by 50 Hz Power line ELF-EMF and the protective effects of Vit C and Vit E.

**Methods:**

We conducted this study in Beijing, China in 2013. B_95-8_ and Balb/c-3T3 cells were divided into a sham group, an expo group and 3 expo groups in which the cells were preincubated with various concentrations of Vit C and Vit E. Then, all of the cells were exposed to 50 Hz Power line ELF-EMF and examined for apoptosis. The cells were collected for apoptosis detection after exposure.

**Results:**

The percent of cells that undergoing apoptosis and preincubated with various concentrations of Vit C and Vit E were significantly lower than in the Expo group.

**Conclusion:**

Vit C and Vit E exert significant protective effects from 50 Hz ELF-EMF radiation. The optimal protective concentrations of Vit C and Vit E are 10 μmol/L and 25 μmol/L, respectively. The protective effect of vitamins was more apparent for Balb/c-3T3 cells than B_95-8_ cells.

## Introduction

The extremely low-frequency electromagnetic field (ELF-EMF) is a type of non-ionizing radiation with low-frequency (<300 Hz), mainly emitted by electric transmission lines and household electronic appliances. People cannot escape ELF-EMF electromagnetic pollution, as its distinctive long wavelength makes ELF-EMF easily bypass barriers and obstructions. Therefore, ELF-EMF has become a worldwide health concern.

ELF-EMF induces transient plasma membrane damage, cellular apoptosis (1) and γ-H2AX foci formation (2). Totally, 50 Hz ELF-EMF was found at a flux density of 1 mT could induce a significant increase of DNA fragmentation (3). One of the most accepted hypotheses is the radical pairs mechanism in which the magnetic field influences the kinetics of reactions with radical pair intermediates and increases the concentration of free radicals in the cells (4). Reactive oxygen species (ROS), including the superoxide anion radical (O_2_^−^·), OH and H_2_O_2_, were assumed to be responsible for the change in mitochondrial trans membrane potential (MTP), apoptosis, γ-H2AX foci formation, and DNA fragmentation. However, the cellular mechanism is still unclear; thus, it is imperative to determine the biological impacts and cellular mechanism of ELF-EMF. In addition, there is an urgent demand for radiation-resistant products. Vitamins are an excellent choice because of their predominant antioxidant abilities, particularly Vit C and Vit E. The ROS scavengers Vit C and Vit E could protect rats from ELF-EMF and electromagnetic radiation (EMR) by improving the antioxidant enzymes activities and decreasing malondialdehyde (MDA) formation (5, 6). In clinical applications, Vit C and Vit E are widely used in ionizing radiation to protect patients from injury caused by X-rays, gamma rays and so on. Vit C and Vit E play excellent protective roles against electromagnetic radiation in mice. Nevertheless, similar research is absent for cells. Therefore, we asked if Vit C and Vit E could protect cells from ELF-EMF injury.

In this study, the B_95-8_ and Balb/c-3T3 cells were divided into a sham group, an expo group and 3 expo groups in which the cells were pre-incubated with various concentrations of Vit C and Vit E. Then, all of the cells were exposed to 50 Hz Powerline ELF-EMF and examined for apoptosis. The 50 Hz power line ELF-EMF biological impact of apoptosis and the protective effects of Vit C and Vit E were examined in this study.

## Materials and Methods

### Reagent Preparation

We conducted this study in Beijing, China in 2013. A Vit C tablet (China Resources Doubled-crane Pharmaceutical Co. Ltd., Beijing, China), containing 0.10 g Vit C, was adequately ground and dissolved in 5.7 mL dd H_2_O. The supernatant was collected after centrifugation with the concentration of 1.0 mol/L. The Vit C solution was filtered and diluted to the working concentrations in the subsequent steps.

A Vit E capsule (The Central Pharmaceutical Co. Ltd., Tianjin, China), containing 0.10 g Vit E, was adequately dissolved in 50 mL ethanol to obtain the Vit E solution with a concentration of 4.8 mmol/L. The Vit E solution was filtered and diluted to the working concentrations in the subsequent experiments.

### Cell Culture

The National Institute donated B95-8 cells, marmoset’s lymphocytes integrated by the Epstein-Barr virus, for Viral Disease Control and Prevention. The suspended B_95-8_ cells were cultured in R-1640 medium (Gibco, Life Technologies, Carlsbad, CA, USA), supplemented with 10% bovine serum (Sijiqing Biological Engineering Materials Co. Ltd, Hangzhou, Zhejiang, China) and 1.0% Penicillin (Hyclone, Thermo Scientific, Logan City, UT, USA), and were incubated at 37 °C and 5.0% CO_2_.

The National Institute donated Balb/c-3T3 cells, embryonic fibroblasts isolated from Balb/c mice, for Viral Disease Control and Prevention, Beijing, China. The adherent Balb/c-3T3 cells were cultured in DMEM medium (Gibco, Life Technologies, Carlsbad, CA, USA), supplemented with 10% fetal bovine serum (Gibco, Life Technologies, Carlsbad, CA, USA) and 1.0% penicillin (Hyclone), and incubated at 37 °C with 5.0% CO_2_. When the cells were 70–80% confluent, the cells were removed with 0.25% (W/V) Trypsin-0.53 mM EDTA (Hyclone, Thermo Scientific, Logan City, UT, USA) solution and inoculated at a concentration of 3–5×10^3^ cells/cm^2^. The Balb/c-3T3 cells were stored in liquid nitrogen vapor phase with complete growth medium supplemented with 10% DMSO (Sigma).

### ELF-EMF Exposure System

The sXc-ELF exposure system (It is Foundation, Zurich, Switzerland), was designed to test extremely low-frequency electromagnetic field exposure mainly emitted from electric transmission lines and household electronic appliances. The two four-coil system, placed inside a mu-metal box, generated the stable and homogeneous electromagnetic field at a range of 3–1250 Hz. The heating system kept the temperature at both waveguides at a stable 37 °C.

### ELF-EMF Exposure Protocol

The B_95-8_ and Balb/c-3T3 cells were cultured in a 37 °C incubator with 5% CO_2_. Cells in the exponential phase were subcultured in 50 mm diameter Petri dishes (BD, Becton Dickinson, NJ, USA) by inoculating 1×10^5^ cells/cm^2^. Each Petri dish was filled with 5 mL of culture medium.

To detect if the Vit C was protective against 50 Hz ELF-EMF, the B_95-8,_ and Balb/c-3T3 cells were divided separately and randomly into 5 groups (Table 1). The sham groups were the negative control groups, not exposed to 50 Hz ELF-EMF radiation. The Expo groups were the groups of cells exposed to 50 Hz ELF-EMF radiation. The cells in the remaining three groups were pre-incubated in culture medium with 2 μmol/L, 10 μmol/L and 100 μmol/L of Vit C for 6 h and then exposed to 50Hz ELF-EMF radiation.

**Table 1:** ELF-EMF Exposure protocol with various concentrations of Vitamin C

**Sham (Control)**	**Expo (Under 50 Hz ELF-EMF Radiation)**
B95-8 cells	B95-8 cells
	B_95-8_ cells +2 μmol/L Vit C
	B_95-8_ cells +10 μmol/L Vit C
	B_95-8_ cells +100 μmol/L Vit C
Balb/c-3T3 cells	Balb/c-3T3 cells
	Balb/c-3T3 cells +2 μmol/L Vit C
	Balb/c-3T3 cells +10 μmol/L Vit C
	Balb/c-3T3 cells +100 μmol/L Vit C

The Expo groups were exposed to a 50 Hz ELF-EMF at a field intensity of 2.3 mT for 16 h. After the exposure, the cells were immediately collected for detection of apoptosis. The apoptosis rates in the different groups were compared to determine the biological impacts caused by 50 Hz ELF-EMF radiation and if the Vit C was protective against the radiation.

Similarly, to determine if the Vit E was protective against 50 Hz ELF-EMF, the B_95-8_, and Balb/c-3T3 cells were divided separately and randomly into 5 groups (Table 2). The sham groups were the negative control groups, not exposed to 50 Hz ELF-EMF radiation. The Expo groups were the cells exposed to 50 Hz ELF-EMF radiation. The cells in the remaining three groups were pre-incubated in culture medium with 10 μmol/L, 25 μmol/L and 50 μmol/L of Vit E for 6 h and then exposed to 50 Hz ELF-EMF radiation.

The expo groups, except for the Sham group, were exposed to 50 Hz ELF-EMF at a field intensity of 2.3 mT for 16 h. After the exposure, the cells were immediately collected for detection of apoptosis. The apoptosis rates in the different groups were compared to determine the biological impacts caused by 50 Hz ELF-EMF radiation and if Vit E could protect cells against this radiation.

**Table 2:** ELF-EMF Exposure protocol with various concentrations of Vitamin E

**Sham (Control)**	**Expo (Under 50 Hz ELF-EMF Radiation)**
B95-8 cells	B95-8 cells
	B_95-8_ cells +10 μmol/L Vit E
	B_95-8_ cells +25 μmol/L Vit E
	B_95-8_ cells +50 μmol/L Vit E
Balb/c-3T3 cells	Balb/c-3T3 cells
	Balb/c-3T3 cells +10 μmol/L Vit E
	Balb/c-3T3 cells +25 μmol/L Vit E
	Balb/c-3T3 cells +50μmol/L Vit E

### Cell Apoptosis Assay

The B_95-8_ and Balb/c-3T3 cells were collected and washed with 0.01 M PBS twice and centrifuged at 1000 rpm for 5 min at 4 °C. The cells were subjected to apoptosis assays with Annexin V-Alexa Fluor 488/FITC-PI (20 μg/mL) (4A Biotech Co. Ltd., Beijing, China). The cell concentrations should be no less than 1×10^6^ cells/mL. The results were measured on a FACS flow cytometer (BD Calibur, Becton Dickinson, NJ, USA) and analyzed using the Mod Fit LT ver. 3.0 software.

### Statistical Analysis

All of data are presented as the mean±STD (standard deviation) and statistically analyzed by SPSS 18.0 (Chicago, IL, USA). Statistical significance was determined by Student’s *t*-test. Differences were significant at *P*<0.05.

## Results

### Fifty Hz Powerline ELF-EMF induced B_95-8_ and Balb/c-3T3 cell apoptosis

**Fig. 1:**
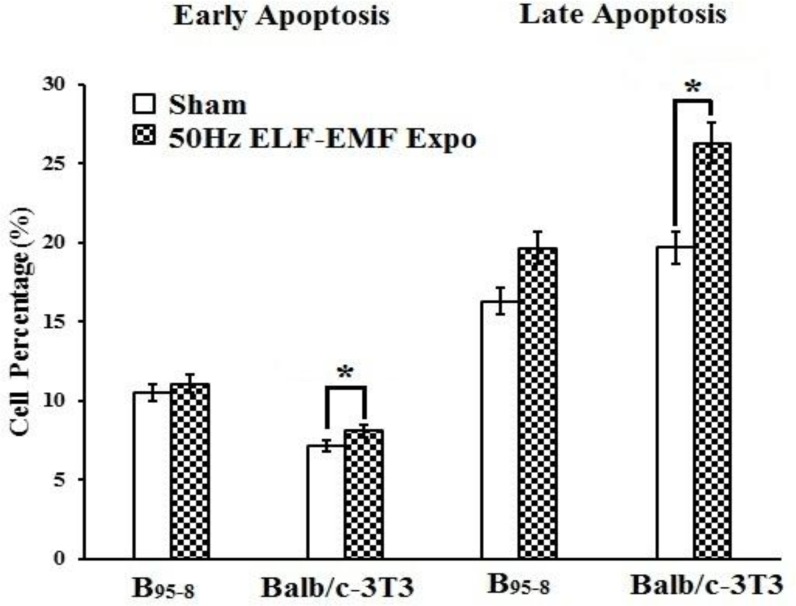
Fifty Hz Powerline ELF-EMF induces apoptosis in B_95-8_ and Balb/c-3T3 cells

After the B_95-8_ and Balb/c-3T3 cells were exposed to 50 Hz Powerline ELF-EMF at an intensity of 2.3 mT for 16 h, both the early and late apoptosis percentages were determined. For B_95-8_ cells, the percent of cells undergoing early apoptosis was slightly higher than in the Sham group, while the percent of cells undergoing late apoptosis was significantly greater than in the Sham group (Fig. 1). For Balb/c-3T3 cells, significant early and late apoptosis were induced in the Expo group due to the 50 Hz Power-line ELF-EMF radiation (*P<0.05) (Fig. 1).

When the B95-8 and Balb/c-3T3 cells were exposed to 50 Hz Powerline ELF-EMF at an intensity of 2.3 mT for 16 h, both the early and late apoptosis percentages increased compared with the sham group. Values are mean ± standard error of mean (SEM). *P<0.05 compared with the Sham group.

### Vitamin C protected B_95-8_ and Balb/c-3T3 cells from early apoptosis induced by 50Hz ELF-EMF

**Fig. 2:**
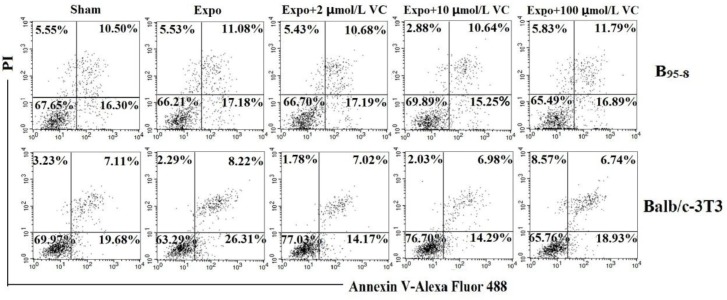
The protective function of Vit C against 50Hz ELF-EMF.

**Fig. 3:**
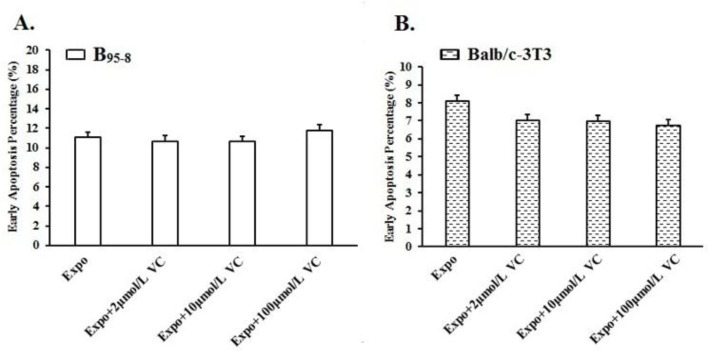
Protective effect of Vit C in B_95-8_ and Balb/c-3T3 cells against early apoptosis.

After 16 h of 50 Hz ELF-EMF exposure, the percentage of B_95-8_ cells in early apoptosis increased (Fig. 2). For the B_95-8_ cells, pre-incubated in the culture medium with 2 μmol/L and 10 μmol/L of Vit C, the early apoptosis percentages decreased to 10.68% and 10.64%, respectively (Fig. 2, Fig. 3A), compared with the Expo group (11.08%). When the cells were cultured in medium with 100 μmol/L Vit C, the early apoptosis percentage was 11.79%, higher than the Expo group (11.08%). The optimum protective concentration of Vit C for B_95-8_ cells was most likely 10 μmol/L.

Compared with the Expo group, a decline in early apoptosis was evident in Balb/c-3T3 cells among the three Expo groups in which the B_95-8_ cells were pre-incubated in medium supplemented with 2 μmol/L, 10 μmol/L and 100 μmol/L Vit C (8.22% to 7.02%, 6.98% and 6.74%, respectively) (Fig. 2, Fig. 3B). In this situation with Balb/c-3T3 cells, the protective function appears stronger with a higher concentration of Vit C.

The flowchart shows the protective function of Vit C for B_95-8_ and Balb/c-3T3 cells, following exposure to intermittent 50Hz Power-line ELF-EMF at a field intensity of 2.3 mT for 16 h. After exposure, both the early and late apoptosis percentages of B_95-8_ and Balb/c-3T3 cells increased compared with the Sham group. The early apoptosis percentages of B_95-8_ cells with Vit C preincubation apparently lowered; however, the late apoptosis percentages did not decrease, except for cells preincubated with 10 μmol/L Vit C. Both the early and late apoptosis percentages of Balb/c-3T3 cells preincubated with Vit C decreased after exposure.

The histogram shows the protection against early apoptosis with diverse concentrations of Vit C in B_95-8_ and Balb/c-3T3 cells. (A). B_95-8_ cell early apoptosis decreased with 2 μmol/L and 10 μmol/L Vit C preincubation but increased with 100 μmol/L Vit C preincubation. (B). Early apoptosis percentages in Balb/c-3T3 cells decreased among the three Expo groups with 10 μmol/L, 25 μmol/L, and 50 μmol/L Vit C preincubation.

### Vitamin C protected B_95-8_ and Balb/c-3T3 cells from late apoptosis induced by 50 Hz ELF-EMF

**Fig. 4:**
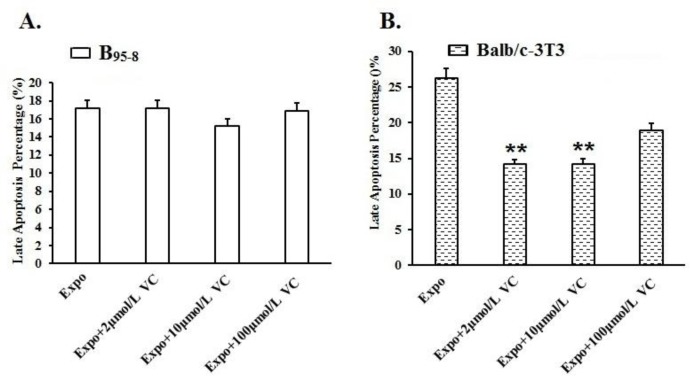
Protective effect of Vit C against late apoptosis in B_95-8_ and Balb/c-3T3 cells.

As shown in Fig 4A, the late apoptosis rates in the B_95-8_ cells preincubated with 2 μmol/L and 100 μmol/L Vit C (Fig. 3, Fig. 4A) were not obviously reduced compared with the Expo group. When the B_95-8_ cells were preincubated with 10 μmol/L Vit C, the percent of cells in late apoptosis apparently decreased (Fig. 3; Fig. 4A). However, the results seemed insignificant.

After 16 h of 50 Hz ELF-EMF radiation, late apoptosis was induced in Balb/c-3T3 cells. In Fig. 4B, all of the late apoptosis percentages in the 3 Expo groups preincubated with 2 μmol/L,10 μmol/L and 100 μmol/L Vit C decreased significantly compared with the Expo group (**P<0.01) (Fig. 3, Fig. 4B). The late apoptosis rate in the Expo group was 26.39%. While, the late apoptosis rate in the Balb/c-3T3 cells preincubated with two μmol/L of Vit C was 14.17%, almost half the Expo group. The late apoptosis percentage in cells preincubated with 10 μmol/L of Vit C was 14.29%. The late apoptosis percentage in cells preincubated with 100 μmol/L of Vit C was 18.39%. The Vit C exerted remarkable protective effects in Balb/c-3T3 cells exposed to 50 Hz ELF-EMF radiation.

The histogram shows the protective effect from late apoptosis in B_95-8_ and Balb/c-3T3 cells preincubated with diverse concentrations of Vit C. (A). The late apoptosis percentages were insignificantly lower in B_95-8_ cells preincubated with 2 μmol/L, 10 μmol/L and 100 μmol/L Vit C. (B) The late apoptosis percentages of Balb/c-3T3 cells preincubated with 2 μmol/L and 10 μmol/L Vit C were significantly lower than the Expo group induced by 50Hz ELF-EMF exposure. The protection of 100 μmol/L VC seemed insignificant **P<0.01.

### Vit E protected B_95-8_ and Balb/c-3T3 cells from early apoptosis induced by 50Hz ELF-EMF

**Fig. 5:**
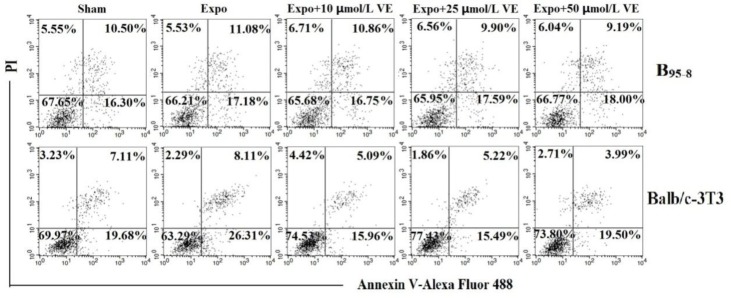
The protective effect of Vit E against 50 Hz ELF-EMF.

**Fig. 6:**
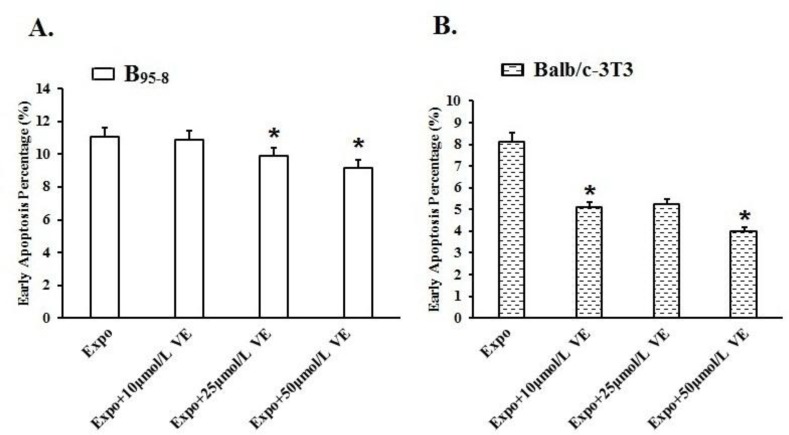
The protective effect of Vit E in B_95-8_ and Balb/c-3T3 cells against early apoptosis.

Fifty Hz Powerline ELF-EMF exposure for 16 h at the intensity of 2.3 mT induced early apoptosis in B_95-8_ cells. The early apoptosis percentages in the B_95-8_ cell preincubated with 10 μmol/L, 25 μmol/L and 50 μmol/L Vit E were, respectively, 10.86%, 9.90% (*P<0.05) (Fig. 5, Fig. 6A) and 9.19% (*P<0.05) (Fig. 5, Fig. 6A), were slightly lower than that in Expo group (11.08%). The early percentages went lower and lower when the cells were preincubated in the medium with higher Vit E.

After 50 Hz ELF-EMF radiation, the early apoptosis rate was 8.11% (Fig. 5), higher than in the Sham group, meaning that 50 Hz ELF-EMF radiation-induced early apoptosis in Balb/c-3T3 cells. The early apoptosis rates in the Balb/c-3T3 cells that were preincubated with 10 μmol/L, 25μmol/L and 50 μmol/L Vit E were 5.09% (*P<0.05) (Fig. 5, Fig. 6B), 5.22% and 3.99% (*P<0.05), respectively (Fig. 5, Fig. 6B).

The flowchart shows the Vit E protective effect in B_95-8_ and Balb/c-3T3 cells after exposure to intermittent 50 Hz Power-line ELF-EMF at a field intensity of 2.3 mT for 16 h. After exposure, both the early and late apoptosis percentages of B_95-8_ and Balb/c-3T3 cells increased compared with the Sham group. The percent of B_95-8_ cells preincubated with Vit E in early apoptosis decreased; however, the percent in late apoptosis did not reduce, except for cells preincubated with 10 μmol/L Vit E. Both early and late apoptosis percentages of Balb/c-3T3 cells with Vit E preincubation decreased after exposure.

The histogram shows the protective effect of various concentrations of Vit E in B_95-8_ and Balb/c-3T3 cells against early apoptosis. (A). The percentage of B_95-8_ cells preincubated with 10 μmol/L and 25 μmol/L of Vit E in early apoptosis significantly decreased but in significantly lowered in the cells preincubated with 25 μmol/L Vit E preincubation. (B). Balb/c-3T3 cells preincubated with 10 μmol/L, 25 μmol/L, and 50 μmol/L of Vit E had obviously decreased percentages of cells in early apoptosis compared with the Expo group *P<0.05.

### Vitamin E protected B_95-8_ and Balb/c-3T3 cells from late apoptosis induced by 50 Hz ELF-EMF

**Fig. 7:**
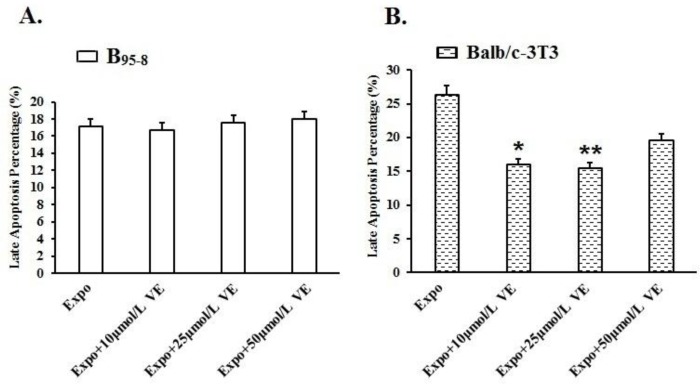
Protective effect of Vit E against late apoptosis in B95-8 and Balb/c-3T3 cells.

After exposure to 50 Hz ELF-EMF radiation, the late apoptosis percentage of B_95-8_ cells increased from 16.30% to 17.18% (Fig. 6). Compared with the Expo group, the late apoptosis percentages in the groups preincubated with diverse Vit E-solutions (10 μmol/L, 25 μmol/L, and 50 μmol/L) were slightly and insignificantly changed after exposure (Fig. 7A, *P*>0.05).

When the Balb/c-3T3 cells were exposed to 50 Hz Power-line ELF-EMF, the late apoptosis rate in Expo group (26.31%) was apparently higher than in the Sham group (19.68%) (Fig. 6). Fifty Hz ELF-EMF induced late apoptosis in Balb/c-3T3 cells. However, the Balb/c-3T3 cells preincubated in medium with Vit E had much lower late apoptosis rates than the Expo group after 50 Hz ELF-EMF radiation.

The late apoptosis percentages in cells with 10 μmol/L and 25 μmol/L Vit E were 15.96% (*P<0.05) and 15.49% (**P<0.01), respectively: much lower than in the Expo group (26.31%) (Fig. 7B). No significant changes were found in the Balb/c-3T3 cells preincubated with 50 μmol/L Vit E.

(A). After exposure, no significant changes were found in B_95-8_ cells, including the Expo group and the 3 groups preincubated with 10 μmol/L, 25 μmol/L, and 50 μmol/L Vit E. The VE protection from late apoptosis induced by 50 Hz ELF-EMF seemed insignificant for B_95-8_ cells. (B). 50Hz ELF-EMF significantly induced late apoptosis in Balb/c-3T3 cells (*P<0.05). Vit E solution with diverse concentration significantly decreased the late apoptosis percentages comparing with Expo group (*P<0.05) (**P<0.01).

## Discussion

ELF-EMF are electromagnetic fields consisting of extremely low-frequency radio waves generated by lightning and natural disturbances in Earth’s magnetic field. ELF frequencies have been used in several manufactured communication systems because of the difficulty of building antennas that can radiate such long waves.

ELF-EMF can be useful in submarine communication systems because ELF-EMF waves can penetrate seawater. Proper ELF-EMFs are widely used in clinical settings. Research has indicated that the specific frequency of ELF-EMF promotes cell proliferation (7), neuronal differentiation (8) and reduces newborn neuron apoptosis (9). 1 Hz ELF-EMF spurred cells to produce protective hydrogen peroxide from DNA damage caused by X-ray irradiation (10). Similarly, heat shock of gene expression can be induced by the proper ELF-EMF for cancer therapy (11). ELF-EMFs have been widely used in genital system wounds (12) and articular cartilage (13) and typhoid therapy (14).

Typically, 50 Hz ELF-EMF is released when electric lines are transmitting and when house-hold electronic appliances are working. The impacts of ELF-EMF to human health have been a research hot topic in recent years since the research (15) indicated a probable association of ELF and childhood leukemia in 1979. To date, much research has begun to determine the biological impacts of ELF-EMFs. ELF-EMF exposure increases intracellular spaces, decreases the numbers of mitochondria (16) and induces transient plasma membrane damage (1). ELF-EMF could affect Na^+^ /Ca^+^ ion channels by increasing Na^+^ concentrations and inhibiting T-type calcium channels via the AA/LTE4 signaling pathway (17). Sixty Hz ELF-EMF inhibited spermatogenesis recovery after reversible testicular damage induced by heat (18). ELF-EMF exposure affected gene expression, including genes involved in metabolic processes, cytoskeletal organization, mitotic spindle organization, cell death, protein modification and proteolysis (19). ELF-EMF has been shown to block cell cycle phases (20), induce DNA damage and γH2AX foci formation (18, 21), and induce oxidative stress (22–24) and cell apoptosis (25, 26). Apoptosis, an intricate cell programmed death involving various genes and proteins, such as Bcl-2, p53, caspase, c-myc and so on, is closely linked to many diseases and has been a research focus in recent years. Because ELF-EMFs are a potential health risk, the identification of radiation-resistant products is an urgent demand. Vitamins are excellent choices for their predominant antioxidant abilities, particularly Vit C and Vit E. Recently, Vit C and Vit E have been widely used in the clinical protection of ionizing radiation than daily protection of ELF-EMF. There are many studies focusing on the protective functions of Vit C and Vit E in mouse models and not in cell models.

In this study, both suspended (B_95-8_) and adherent cells (Balb/c-3T3) were divided into a Sham group, an Expo group and 3 Expo groups in which the cells were preincubated with diverse Vit C and Vit E concentrations and exposed to 50 Hz Power-line ELF-EMF for 16 h at an intensity of 2.3 mT. After exposure to 50 Hz Power-line ELF-EMF, obvious increases in early and late apoptosis in B_95-8_ and Balb/c-3T3 cells were observed. The percent of the cells undergoing apoptosis preincubated with various Vit C and Vit E concentrations were obviously and significantly lower than in the Expo group. Therefore, Vit C and Vit E exert significant protective effects from 50 Hz ELF-EMF radiation. The optimal protective concentration of Vit C and Vit E are 10 μmol/L and 25 μmol/L, respectively. The protective function of vitamins was more apparent for Balb/c-3T3 cells than B_95-8_ cells.

Although the mechanism of ELF-EMF biological impacts remains unclear, it is hypothesized that free radicals are involved. ELF-EMF induces oxidative stress, increases in ROS and lipid peroxide and a decrease in antioxidant enzyme activity. Reactive oxygen species (ROS) encompass a variety of diverse chemical species including superoxide anions (O_2_·^−^), hydroxyl radicals (·OH) and hydrogen peroxide (H_2_O_2_). These various radical species can be generated exogenously when the cells are under external stimuli and endogenously in the mitochondria. The burden of ROS production is mainly annihilated by an intricate antioxidant defense system that includes the enzymatic scavenger’s superoxide dismutase (SOD), catalase (CAT) and glutathione peroxides (GPx). The balance between ROS production and antioxidant defenses determines the degree of oxidative stress (27). The oxidative stress that is not scavenged by antioxidant defenses can induce cell apoptosis, lipid peroxidation, DNA fragmentation, and affect cell signal transduction via p53, PI (3) K-Akt, JNK, and NF-kB pathways, etc.

Vitamins are natural free radical scavengers, particularly Vit C and Vit E (28, 29). Vit C, a water-soluble free radical scavenger, can produce half-dehydrogenated Vit C after donating a hydrogen atom and produce dehydrogenated Vit C (Vit·C) after donating two hydrogen atoms. The free radicals are scavenged by a hydrogen atom donated by Vit C, which immediately reacts with O_2_·^−^, ·O and H_2_O_2_. The Vit·C is recovered to Vit C by gaining hydrogen atoms from NADPH. In addition, Vit E is another efficacious free radical scavenger by scavenging O_2_·^−^ and unsaturated fatty acid peroxide radicals (RO_2_·^−^) and by quenching O_2_·^−^ from cell membrane attacks. Vit E can be recovered to Vit E in the presence of a high concentration of Vit C. Vit C and Vit E can scavenge free radicals to decrease the concentration of glutathione and glutathione peroxidase and decrease the production of malonaldehyde. Vit C activated tumor suppressor genes by demethylation to protect cells from becoming tumor cells (30). In this study, ROS formed when the cells were exposed to 50 Hz ELF-EMF. The ROS burden eliminated by anti-oxidant enzymes induced apoptosis. When the cells were preincubated with diverse concentrations of Vit C and Vit E, Vit C and Vit E. scavenged the un-cleared ROS. As a result, the apoptosis of B_95-8_ and Balb/c-3T3 cells was substantially lowered. The optimal protective concentration of Vit C and Vit E is hypothesized to be 10 μmol/L and 25 μmol/L, respectively.

In this study, the protective function of Vit C and Vit E for Balb/c-3T3 cells was better and more significant than for B_95-8_ cells. When the B_95-8_ cells were cultured, the suspended cells preferred to cluster and form a large “cell ball”. When the cells were exposed, the inner cells were covered and protected by the outer cells from ELF-EMF radiation. The cells may communicate with one another by cell communication systems via chemical signal secretion and contact-dependent signaling. However, the outer suspension cells were still under greater exposure than the inner cells. Epidermal cells are more inclined to be affected by ELF-EMF radiation and therefore absorb more ELF-EMF than inner cells of the tissues. It would be meaningful to choose susceptible target cells and tissues. The adherent cells are more likely to obtain uniform and equal exposure. Therefore, adherent cells are more appropriate for researching the mechanisms of ELF-EMF biological effects.

## Conclusion

Vit C and Vit E exerted significant protective effects from oxidative stress induced by 1h intermittent 50 Hz ELF-EMF radiation. The optimal protective concentrations of Vit C and Vit E are 10 μmol/L and 25 μmol/L, respectively. The protective effect of vitamins was more apparent for Balb/c-3T3 cells than B_95-8_ cells.

## Ethical considerations

Ethical issues (Including plagiarism, informed consent, misconduct, data fabrication and/or falsification, double publication and/or submission, redundancy, etc.) have been completely observed by the authors.
